# Understanding the learning experience of Chinese nursing students in an English-medium instructional program: growth and challenges

**DOI:** 10.1186/s12909-025-07966-2

**Published:** 2025-10-09

**Authors:** Qianyu Liang, Jiaqi Zheng, Weichen Zhang, Qingxian Liu, Xiaodan Lin, Miaolan Guo, Jing Su

**Affiliations:** Department of Nursing, Shantou University Medical College, Shantou, 515041 China

**Keywords:** Nursing education, Undergraduates, English-medium instruction, Qualitative research, Educational reform

## Abstract

**Background:**

English has served as the predominant language for academic communication on a worldwide scale. There is a growing demand for nursing professionals that possess international competency. However, the experience of utilizing English as a medium of instruction (EMI) for teaching nursing academic courses in mainland China is inadequate.

**Purpose:**

In depth explore the Chinese nursing students’ learning experience under EMI teaching mode.

**Methods:**

Thirty-two undergraduate students from Nursing EMI program were selected using convenience sampling to participate in Focus Group Discussion (FGD).

**Results:**

Three major themes have been extracted: advantages of English Medium Instruction, challenges encountered during the process of EMI learning, students’ suggestions on Nursing EMI program. These three major themes encompass fifteen sub-themes.

**Conclusion:**

The Nursing EMI program has improved students’ comprehensive professionalism and international competency, however, coping with using English as the medium of instruction in China’s Nursing education remains in exploratory stage, and requires further educational reform.

**Supplementary Information:**

The online version contains supplementary material available at 10.1186/s12909-025-07966-2.

## Introduction

The general internationalization trend has underscored the imperative to improve the quality of Nursing education, cultivate nurses that are interculturally competent, and ultimately propel the actualization of global health [1–4]. As the most pivotal information carrier, English has been the dominant language in the globalized medical realm [5, 6], where substantial academic texts, sources and materials are presented in English [7], which is of paramount significance for the implementation of Evidence-Based Practice in Nursing.

Driven by global imperatives, the adoption of English as a Medium of Instruction (EMI) by higher education institutions (HEIs) in non-English-speaking countries has become an increasingly prevalent trend worldwide [8]. Over the past few decades, universities in European countries such as Germany [9] and Netherland [10] have promoted the English-medium programs extensively, followed by universities in Asian regions such as Korea [11], Vietnam [12] and Taiwan, China [5], which have dramatically increased the number of English-medium courses. With the rapid evolution of society and growing recognition of Nursing [13], EMI courses of Nursing have successively been introduced in Mainland, China, for example, the Bilingual Nursing Program of Guangzhou Medical University, where academic knowledge is delivered in both English and Chinese. In 2013, an EMI program called “All-English Program Nursing” was established in X(referring to a specific University Medical College) aimed at training a cohort of excellent nursing professionals who can adapt to the rapid advancements in medical and nursing sciences and technology, utilize English to master professional knowledge, and have the potential to advance the internationalization of China’s nursing discipline.

Whereas the adoption of EMI in medical and nursing education has gained worldwide momentum, significant discrepancies regarding the selection of instructional language—specifically whether to use English or native language—have been revealed in exiting research [14–16]. Geographically, while a significant number of nations prioritize instructions using native language, areas including India and Hong Kong have adopted English as the main medium of all medical courses [14]. Even in regions where EMI is widely implemented, the perspectives of various stakeholders exhibit considerable differences. In detail, most policymakers and instructors consistently demonstrate strong support for EMI in medical curricula, as they regard English as a pivotal tool to enhance international competitiveness of the institution, and further promote students’ career advancement [15, 17]. In contrast, students’ opinions display a more evident ambivalence, reflecting practical concerns closely related to their individual development. This ambivalence is echoed in previous studies, which have shown diverse and sometimes contradictory findings regarding EMI in the healthcare field, including both positive and negative impacts and attitudes, as well as differing preferences for monolingual versus bilingual instruction [18]. It is widely acknowledged by most students that EMI programs have offered substantial advantages, encompassing improvements in English proficiency [17, 19] as well as learning ability [20], and increased access to cutting-edge medical resources [21]. Notwithstanding, challenges encountered by students in the learning process should not be overlooked. Some students noted that instruction through English has created obstacles in understanding professional concepts [14–16], demanding extra time and effort to achieve satisfactory academic results [14, 22] which has in turn contributed to adverse psychological impacts [23, 24].

Existing studies on medical EMI programs predominantly focuses on students’ attitude, with quantitative approaches such as questionnaire, constituting the largest proportion of research design. However, the subjective learning experiences of EMI students remain significantly understudied. In particular, qualitative research on nursing students’ attitude and perception toward EMI programs in the academic context of Mainland, China is profoundly scarce, representing a critical research gap.

This study seeks to address this gap by exploring the learning experiences of undergraduate nursing students enrolled in an EMI program in Mainland China. Through qualitative focus group discussions, we aim to uncover how students perceive the benefits and challenges of EMI, how it shapes their academic and professional development, and what improvements they suggest for future program optimization. By capturing students’ voices, this research offers grounded insights to complement the existing research structure, and to further investigate the disputes over medium of instruction selection, grounded in students’ perspectives.

## Participants and methods

### Research participants

Using convenience sampling, 32 undergraduate students from Nursing EMI Program in X(referring to a specific University Medical College) were recruited as the participants of this study. Inclusion Criteria: (1) Undergraduate students from Nursing EMI program in X(referring to a specific University Medical College) (2) participants who provided informed consent. Exclusion Criteria: (1) Students who were suspended from school (2) Participants who dropped out before the study commenced. Focus group interviews were chosen to collect data. The interactive nature of focus groups can encourage participants to elaborate on their responses based on the viewpoints expressed by peers, thereby enriching the data with diverse perspectives and reinforcing the validity of the findings. This method was suitable for understanding the shared experiences of students in the Nursing EMI program. The sample size was determined based on data saturation, which occurs when no new codes or themes emerge from the data. In this study, saturation was evaluated through the ongoing comparison of emerging codes during concurrent data collection and analysis. After the sixth focus group, no additional substantive themes were identified. The determination of saturation was made through team discussion and consensus among all members of the research team. For the sake of confidentiality, potentially identifiable information was not recorded and each participant was assigned a code number from N1 to N32. Included participants were divided into 6 focus groups, Focus Group A – C: 4 students each group, Focus Group D – F: 7 students each group.

### Research methods

#### Identifying the interview outline

The development of the interview guide followed a structured, multi-step process. An initial draft was formulated through discussions within the research team, based on the research objectives and existing literature. The preliminary guide was then reviewed by experts in nursing education and faculty members involved in EMI course delivery to evaluate the clarity, relevance, and appropriateness of the questions. Following expert review, two undergraduate students with experience in EMI programs were invited to participate in pilot interviews. Based on their feedback and the observed flow of conversation, minor adjustments were made to the wording and sequencing of the questions to enhance clarity and contextual sensitivity. The finalized version of the interview guide was then used consistently across all focus groups, as follows: (1) What kind of experience do you think it is to learn in the Nursing EMI program? (2) Through Nursing EMI learning, in what aspects you got improved? (3) In your mind, what are the difficult parts of Nursing EMI learning? (4) What aspects of the Nursing EMI program do you think can be improved? The interview guide used in this study was specifically developed for this research. The complete English version of the interview guide is provided as a supplementary file (see Supplementary File 1).

#### Data collection

Between January 20th to February 8th, 2024, group-focused, semi-structured interviews were conducted by one chief researcher who had received professional training and other two assistant researchers. Interviews were performed in a conference room, guaranteeing the environment were quiet, comfortable, and free from disturbance. A semi-structured interview schedule was used to collect data. Before the interviews, the schedule was organized based on the interviewees’ preferences. Additionally, all participants were informed of the purpose and duration of the interview and agreed to the recording. The interviews were conducted in Mandarin Chinese. At the beginning of each interview, the interviewer will explain the main content of the interview. Afterwards, discussions oriented towards the outline of the interview began. During the 60-to-90-min interview for each group, interviewers had to continually observe and mark down the participants’ facial expressions and emotional changes, while contemporaneously collecting information, and their feelings and attitudes had to be clarified and confirmed. Under the premise of no digression derived, every participant was encouraged to express opinions. The audio recordings were initially analyzed in Chinese, and subsequently, the findings were translated into English for further analysis and reporting. The translation process involved senior nursing education experts and native English-speaking educators for quality control, ensuring the accuracy of the translated content.

#### Data analysis

Colaizzi’s phenomenological method was used to analyze participant transcripts. Researchers first read all transcripts repeatedly to obtain a holistic understanding of the data. Significant statements related to participants’ experiences with the EMI program were then extracted from the transcripts. These statements were interpreted to formulate meanings, a process conducted independently by researchers with varying degrees of proximity to the EMI experience. Microsoft Word was used to assist in data organization, including sorting, annotation, and coding. The formulated meanings were grouped into clusters of themes, allowing shared patterns to emerge across participants’ experiences. These themes were then synthesized into a comprehensive and exhaustive description of the phenomenon. Subsequently, the fundamental structure underlying participants’ experiences was identified. Finally, the results were returned to the participants for member checking to enhance the credibility of the study. To ensure inter-coder reliability, independently coded data were cross-checked by multiple researchers. Any discrepancies in interpretation were discussed and resolved through team consensus, enhancing the consistency and rigor of the coding process.

### Trustworthiness

To ensure the trustworthiness of this study, we followed Lincoln & Guba’s criteria of credibility, transferability, dependability, and confirmability [25]. Credibility was achieved through several strategies: a purposive sampling facilitated a detailed and nuanced account of the EMI program experiences, ensuring that the findings were grounded in the direct accounts. Member checking involved presenting a summary of key findings to participants. Experienced nursing experts reviewed the study to ensure analytic rigor and alignment with the phenomenological approach. Secondly, transferability was supported by detailed information on the data collection method, period, and participants’ characteristics. Third, dependability was established through an audit trail, cross-checking between members of the research team until consensus was reached, and accurate documentation of all processes. Finally, confirmability was achieved through systematic preservation of all data, such as recordings, verbatim transcripts, reflective daily records and analyzed records, following established research standards.

In terms of research team composition, the team consisted of nursing and medical faculty members, a postgraduate nursing student, and individuals with firsthand EMI learning experience. All team members had received formal training in qualitative research, ensuring a shared foundational understanding of methodological principles and analytic rigor. This multidisciplinary composition provided a range of complementary perspectives that enriched both data collection and interpretation. Faculty members contributed expertise in nursing education and curriculum design, while the postgraduate student brought relevant pedagogical insights through her experience as a teaching assistant in nursing courses. Although she did not have personal EMI learning experience, her familiarity with the course content contributed to balanced, informed analysis. Researchers with EMI learning backgrounds offered insider perspectives that enhanced the contextual relevance of the findings. To minimize bias, researchers engaged in bracketing by identifying and setting aside their own assumptions throughout the research process.

## Results

Based on the analysis of the focus group data, three overarching themes were identified, each encompassing multiple sub-themes that reflect different aspects of students’ experiences in the Nursing EMI program. These themes include: (1) Advantages of English Medium Instruction, (2) Challenges Encountered During EMI Learning, and (3) Students’ Suggestions on the Nursing EMI Program.

**Fig. 1:**
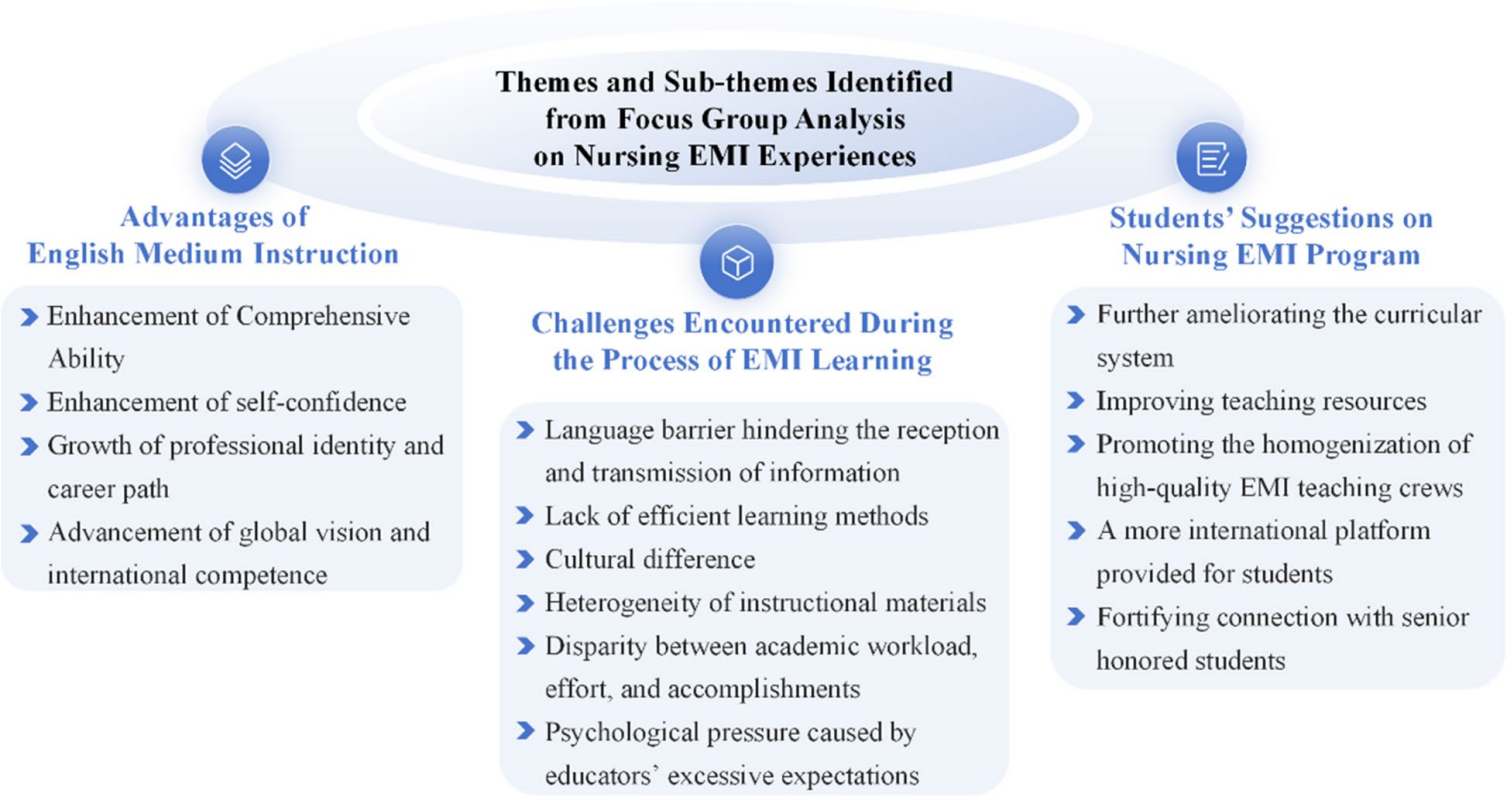
Themes and sub-themes identified from focus group analysis on nursing EMI experiences

A total of fifteen sub-themes were distilled from the data, providing nuanced insights into students’ perceived academic and personal growth, encountered difficulties, and proposed improvements. To enhance clarity and facilitate understanding, a summary figure (Fig. 1) is presented below to illustrate the thematic structure and its associated sub-themes.

### Theme 1: Advantages of English medium instruction

#### Enhancement of comprehensive ability

##### Development of self-directed learning ability

A core advantage of the EMI program in enhancing students’ comprehensive competences lies in its facilitation of a shift from passive knowledge reception to active learning, ultimately fostering robust self-directed learning ability. The shift is driven by factors including the altered learning requirements, innovative teaching strategies, and a motivating peer environment, all of which are reflected in students’ lived experiences.

To begin with, students’ learning approach has shifted from a more passive and receptive mode to a more active and engaged one, to meet the knowledge requirements and keep up with the pace of class. Over time, students began to probe into the key points of each course, and spontaneously search relevant information concerning what they showed interest in.

One student(N8) shared her insights into the changes in her learning initiative, noting “I think I’ve been in a passive learning state since high school, meaning I accepted and followed whatever teachers told me, but after enrolling in the EMI program, because of changes in the teacher’s requirements and learning objectives, I gradually adapted to initiative learning, rather than just accepting. Now I actively search and study in depth the knowledge I’m interested in”.

Beyond the classroom, the demand for active engagement extends to review after class, which has been claimed to be an indispensable step to compensate for the linguistic and cognitive challenges in absorbing the content. N9 highlighted, “Proactive learning after class was necessary because EMI learning was challenging and difficult to comprehend thoroughly in class.” The after-class consolidation is a strategic in response to EMI’s demands, training students to identify their own knowledge deficits and address them independently.

In addition, EMI courses adopting innovative teaching strategies such as Flipped Classroom and Problem-Based Learning (PBL) further reinforces the active engagement and central role of students in their learning processes, where students are required to study and think centered on cases and problems. N4 elaborated on the impact of PBL classes, stating “Just like dealing with actual patients, in PBL lessons, you had to obtain information from the given cases, analyze relevant test results, point out existing problems, make diagnoses, and propose solutions, using what you have learned to solve problems. After class, you had to do research, filling in the gap of the knowledge that you had not mastered, thus you had improved your ability of self-learning.” Such student-centered simulations on largest extent helped students exert their subjective initiatives, and improve their ability of independent learning, comprehensive analysis, and creativity.

Finally, the positive atmosphere of the class serves as a critical sustaining support, prompting students to maintain as well as further intensify their learning initiative. N9 stated: “EMI students all are excellent learners, prompting you to proactively improve yourself, and if you don’t work hard enough, you’ll be left behind, so you must be better prepared for each learning task.”

##### Improvement of academic English

As one of the prominent manifestations of EMI students’ enhancing competences, the academic English proficiency has been developed throughout the learning process, from mastering basic language skills to corresponding application in academic tasks.

EMI courses provide a context-rich learning environment that accelerates the mastery of core language skills, such as the expansion of medical English vocabulary and the improvement of English comprehension. N2 noted, “To master medical vocabularies was very challenging, but now we have made elementary acquaintance with them. I did not realize this until I took an IELTS test, the accumulation of medical terms during the EMI courses has made it less time-consuming to memorize the IELTS vocabulary.” The reflection of N9 aligns with N2, stating, “Extensive reading and listening tasks in class have improved our vocabulary and listening comprehension imperceptibly.” Taken together, these accounts show that the repeated exposure to professional English in class not only strengthens vocabulary but also makes language learning an implicit and continuous process rather than a separate task.

With fewer restrictions from language barriers, EMI students have become more fluent in retrieving English materials and literature, through which a scientific research mindset has been developed gradually. N10 emphasized this, explaining “With English as the sole medium to study, I had to search for international literature instead of limiting myself to Chinese literature, throughout this process my literature retrieval skills have been fortified.” In this way, academic English proficiency becomes a “bridge” connecting basic language skills and advanced academic competence. Student N5 captured this, stating “More importance has been attached to the enhancement of scientific research ability in the EMI program. We gradually developed a mindset for scientific research and took the initiative to undertake research projects.”

##### Strengthening resilience in stress management and problem solving

EMI students face with a more unfamiliar learning environment, that is, using English as a Second Language (ESL), where they have to devise learning approaches independently to overcome challenges. N4 stated: “Studying and taking exams in English, our academic pressure was much higher compared to using Chinese. To conquer the language barrier, we had to make substantial effort including adjusting our mental attitude while studying assiduously. During this process our stress resistance improved.”

N10 further expanded on the constructive role of the stress from EMI program, noting “It is undeniable that the academic pressure under EMI was considerable, but I believe it can be an extrinsic impetus of motivating us to learn and develop better strategies to solve the problems we face.” EMI students have realized their gains during the process of seeking solution and adjusting negative emotion, which contributed to the improvement of their confidence and problem-solving ability, forming a positive feedback loop.

##### Cultivation of leadership, communication and teamwork skills

EMI program’s emphasis on group tasks serves as the foundational platform for developing leadership, communication and collaboration abilities. N9 stated “The EMI courses included a significant number of daily group assignments, mirroring teaching methods used in foreign countries. These assignments have enhanced our abilities in various areas, particularly teamwork.”

Notably, these group tasks also create opportunities for role differentiation, where certain students take on organizational responsibilities. N7 reflected on her experience: “As a project leader, I strove to organize our group, distribute tasks, communicate timely, and finally we completed the task successfully, which made me feel that I have become independent.”

##### Development of critical thinking and clinical reasoning

Students have significantly improved their ability of critical thinking and clinical reasoning in lessons of involving scenario simulations, case discussions and hospital visits, where they combine the theoretical knowledge with clinical scenarios, ponder over the whole process of disease occurrence and development, oriented by cases, and analyze problems from multiple perspectives, critically regard the information presented to them, and identify the nursing problems as well as targeted nursing interventions for patients. Specifically speaking, N3 stated: “I think my clinical reasoning has been enhanced. Now I can integrate the patient’s basic condition, past history, and current medical history, etc., and determine his existing problems, and then propose targeted nursing interventions.”

Moreover, the program’s requirement to use English urges students to engage with a wider range of materials, such as international textbooks and overseas literature. N1 noted, “As for some textbook knowledge which I did not agree with or understand, I would review literature from both domestic and foreign sources to have a better understanding, so as to make better judgement.” This highlights how increased access to abundant literature contributes to their elevated awareness of evaluating the textbook content critically, and ultimately strengthens the quality of clinical reasoning.

#### Enhancement of self-confidence

A significant psychological outcome of EMI participation for nursing students is the noticeable boost in self-confidence, which is driven by the enhancement of students’ English proficiency, increased opportunities for self-expression, and the title “EMI student”.

The first driver of confidence enhancement derives from EMI’s role in developing students’ English proficiency and creating conducive environment for application, as N6 stated, “Thanks to EMI courses, my spoken English has been practiced, and I have become more poised and confident when communicating with others in English. Additionally, there were a lot of opportunities to show ourselves in EMI courses, such as group presentations, which have boosted my confidence in participating in activities such as speech competitions.”

While improved proficiency and increased opportunities build confidence from within, the positive social perception of the identity “EMI student” reinforces the confidence from the outside, further strengthening their self-image. N10 highlighted, “When others heard about the Nursing EMI program, they were convinced that EMI students must be excellent. Many teachers held us in high regard, which effectively boosted our confidence.” Compliments from teachers urge students to elevate their self-expectation and self-demand, to meet the teachers’ appraisement, students have to devote more effort to their study, which fortifies their learning autonomy and students acquired confidence in the process of refining their knowledge and capability.

#### Growth of professional identity and expanse of career path

EMI’s lecturers are mainly highly-educated elites in nursing who impart advanced knowledge and broaden students’ views, and their experience studying abroad also boosts students’ professional identity. Besides, the title of the “EMI student” garner them more encouragement and recognition from others, which has strengthened students’ professional identity and faith in the prospect of Nursing profession.

N8 highlighted the inspirational impact of these instructors, stating “ Inspired by the high-achieving EMI teachers, I became convinced that our developmental path would be broader in the future, and nursing students’ sense of professional identity would subsequently be improved.”

Similarly, N12 also clarified: “The teachers I came into contact with in EMI courses were positioned at a relatively higher level in this field, which made me realize that careers for Nursing students can take various directions, and this has reinforced my sense of professional identity.”

#### Advancement of global vision and international competence

In the Nursing EMI program, foreign instructional educators and international exchange projects have facilitated students’ insights into the professional advancement worldwide and learning conditions of Nursing students at a global extent.

N9 vividly articulated the risk of lacking global exposure, stating: “We need an approach like the Nursing EMI program to provide us with insight into what advanced Nursing knowledge and theories are, otherwise, we would have been stuck with Chinese perspectives, or rarely get to know those in foreign countries, which would hinder the development of the industry.”

N11: “Now I have more confidence in applying for further study or employment abroad. If I had not joined the Nursing EMI program, I would never have had this idea.” The Nursing EMI program has helped the students absorb internationally advanced knowledge, thinking, and widen their global vision, which was conducive for our domestic Nursing to meet with the international standard, and prevented stagnancy.

### Theme 2: Challenges encountered during the process of EMI learning

#### Language barrier hindering the reception and transmission of information

For certain teachers and students with weaker English proficiency, using English as an instructional medium might lead to ineffective knowledge transfer, thus impacting teaching effectiveness. After class, students need to spend considerable time repeatedly consolidating the knowledge, which lowers the learning efficiency to a significant extent, exerting detrimental impact on students’ enthusiasm for studying.

N8 vividly quantified the gap, stating: “For students in Nursing non-EMI program, I think, 70% of the knowledge was absorbed during class, but maybe only 50% for students in the EMI program. After class, we needed more time to fill in the knowledge gap. Lessons taught by foreign teachers were even more challenging, because they tended to teach in a more divergent way, thus requiring more time to grasp the key points.”

#### Lack of efficient learning methods

Early on, students lacked efficient learning methods to cope with the transition of the instructional paradigm from Chinese to English, making it difficult to build a knowledge framework and understand the connotation of knowledge. Students spent such a tremendous amount of time as to comprehend the profound knowledge. However, the exam score they obtained was not equivalent to their effort. Majority of students noted that they did not gradually master their own effective learning method until the second semester of sophomore year, and began to get accommodated to the EMI mode based on accumulation of vocabulary and professional knowledge.

N1: “Basically only if I spent a long time previewing the vocabulary before class, was I able to understand what the teacher said in class, but it was still difficult to memorize words that were too complicated.” The student has identified the vocabulary preview as a basic requirement, but lacks a method to retain complex terms, which turns a necessary preparation step into a time-consuming and frustrating process that fails to solve the understanding problem in class.

Besides the vocabulary challenge, there is a difficulty in knowledge integration. N4 elaborated on this, stating: “When I saw the English slides for the first time, it was extremely difficult for me to understand. I needed to use Chinese textbooks alongside them, but I didn’t know how to connect the knowledge points, so it was a bit strenuous at the beginning.” This disconnect means they cannot organize new information into coherent frameworks, leading to inefficient learning and academic performance below expectations.

#### Cultural difference

Although the same curriculum is adopted by both foreign and Chinese teachers, biases are inevitable due to different cultural backgrounds and instructional methods, which impedes the comprehension of EMI students. Additionally, students admitted that they sometimes failed to understand the questions posed by foreign teachers in the final examination, which has imposed certain pressure on students.

The most direct impact of cultural differences manifests in assessment scenarios, where foreign instructors’ unique thinking and question-design patterns clash with students’ familiarity with the traditional logic in Chinese exams. N3 stated: “The foreign teachers’ way of thinking was notably different from ours, and the way of setting questions was also different, so we could hardly understand the questions on the exam paper. Since foreign teachers adopted the overseas educational methods, while we had been receiving Chinese education since childhood, this was tricky.” This mismatch leaves students struggling to interpret exam questions, even if they have mastered the content, directly affecting their performance and adding academic pressure.

Beyond examinations, cultural differences also shape classroom interactions. N6 noted: “The foreign teachers encouraged us to communicate actively, but we tended to have a relatively superficial understanding of what they taught.” Consequently, even with active interaction, the depth of comprehension was limited.

#### Heterogeneity of instructional materials

Students asserted that the teaching materials for the Nursing EMI program lacked standardization and consistency. Specifically speaking, Chinese teachers use both Chinese and English versions of textbooks as references, while foreign teachers tend to adopt English textbooks and foreign research. At the end of semesters, students find it difficult to effectively make proper use of the materials for purposeful reviewing.

N13 directly articulated this confusion, stating: “The teaching resources seemed to be inconsistent, some teachers referenced the Chinese textbooks, while others referred to different English materials, they did not reach a consensus, so I didn’t know which book to use for review.”

In addition, the lack of relevant exercises or quizzes for knowledge consolidation often left students uncertain during exam preparation. N3 noted: “Without exercises to reinforce my memory, I would still be confused when faced with the exam questions.”

#### Disparity between academic workload, effort, and accomplishments

A core frustration for nursing EMI students lies in the imbalance between the heavy academic workload, significant effort invested, and the outcomes they achieved, especially when pursuing personal aspirations like further education. Contradictory dissonance derived from two existing phenomena: one was the disparity between the unsatisfactory academic marks and massive endeavor students have made, and the other was the incongruity between English-medium instructional paradigm and the necessity of passing the national licensure examination in Chinese.

EMI students spend far more time addressing hurdles such as language barriers, knowledge integration, and cultural differences than non-EMI peers, while this extra effort rarely translates into advantages for further education. N10 revealed: “Admittedly, if I could study abroad in the future, I would adapt faster. However, with lower a Grade Point Average (GPA), it would be exceedingly difficult to apply for colleges or universities overseas. In such cases, studying in the non-EMI program for higher scores might have been better.”

Furthermore, the heavy workload under EMI program also limits students’ availability for pursuing cross-disciplinary further education. N1 explained: “Even if you want to apply for a cross-disciplinary master’s degree, it would be simpler if you were a non-EMI student, at least you can spare part of your time to learn the major you desire for, which few EMI students are able to do.”

Beyond disparity between grades and time, the content taught in EMI program often does not completely align with the requirements for domestic critical exams. N5 clearly identified this issue, noting: “The knowledge involved in the Postgraduate Entrance Examination in China is different from what we learned in EMI courses.” This means EMI students’ effort to master English-medium content becomes a separate test rather than a foundation for subsequent key exams.

#### Psychological pressure caused by educators’ excessive expectations

Some people were skeptical about the necessity of developing Nursing EMI program, meanwhile, their expectations of EMI students were excessive. Students believed that their abilities were likely to fall short of these expectations, which dampened their enthusiasm. N8: “Pressure also came from others’ expectations. Most teachers thought EMI students’ oral English should be superior, surpassing our current level. This mismatch stressed us out.” N10: “It was believed that the Nursing EMI students were outstanding, mastering professional knowledge more deeply. However, compared to non-EMI students, there’s still room for improvement.”

### Theme 3: Students’ suggestions on nursing EMI program

#### Further ameliorating the curricular system

##### Clarifying the cultivation objectives

The teaching goals of the Nursing EMI program should be better clarified, oriented by students’ career development in the future, to train Nursing EMI students in a better planned and targeted manner. N1: “I think it is important to define clearer program objectives before proceeding with the continued development of the Nursing EMI program. Some students will stay in China to practice, while others want to move abroad for further studies. The school should offer additional courses to respond to diverse requirements.”

##### Suggestions on restructuring curriculum

Students have proposed several recommendations for curriculum restructuring, including the integration of professional courses and the retention of courses that emphasize critical thinking skills and professional medical terminology in English to facilitate the transition from Chinese to English language learning.

N14 explicitly proposed the integration for core and ethical courses, stating: “I think that internal and surgical nursing should be better integrated, and that some of the medical ethics class could be better embedded into the practical nursing training.”

Another critical suggestion focuses on addressing the root of language barriers, embedding the medical English terminology into the systematic EMI curriculum. N12 put forward a specific and progressive implementation plan, noting: “The course “PMED” employed innovative instructional methods to teach knowledge on English vocabulary, specifically focusing on roots and affixes. This effectively prepared students with fundamental understanding of Medicine. I suggest that this subject be incrementally included into the Nursing EMI curriculum, starting from year 1 and progressing to year 3. This will enable us to get a comprehensive understanding of medical terminology.” This emphasized that structured terminology training would help ease the transition to English learning from Chinese, and lay a solid foundation for understanding professional content.

##### Modifying the assessment indicators

The students reckoned that the school should formulate a set of assessment standards and separated ranking system exclusively intended for the Nursing EMI students, and the content of the examination should be more congruent with the teaching goals. Diversified assessment modalities including both summative and formative evaluation should be better used to assess students’ performance, and group assignments and presentations should be applied to regularly and timely familiarize the instructors with the teaching effectiveness.

N10: “Students in the EMI program study and take examinations in English, which makes learning more difficult than for students in regular Chinese program. However, when scholarships were awarded, we were all ranked together, which put a lot of pressure on me because I had to work much harder to get one. I think we should be evaluated separately from students who are not in EMI, or we should be given a weighted number depending on how difficult the examinations were.”

Beyond fairness, students also suggest enriching assessment methods to move beyond over-reliance on summative evaluations such as final exams. N20 emphasized: “It is my opinion that the formative and summative evaluations for each course could be better developed, taking into consideration students’ learning burden holistically.”

#### Improving teaching resources

The Nursing EMI students should be provided with more teaching resources, such as foreign scientific journals, websites of teaching videos in both Chinese and English, etc., which can encourage students to learn independently.

N15 highlighted the need for resource accessibility, stating: “I think more foreign books and websites of good courses overseas should be offered, just like some online learning platforms in China. We would like to know more about how to access these resources.”

While expanding international resources is critical, students also emphasized the need for Chinese-medium support resources. N16: “I think tutorial videos of some professional courses in Chinese should be added, so that we can preview the basic knowledge before class, in this way, we can have a better grasp of knowledge in EMI courses.” This not only improves the pre-class preparation efficiently but also directly enhances in-class comprehension, creating a more effective learning cycle.

#### Promoting the homogenization of high-quality EMI teaching crews

The majority of EMI course instructors are Chinese and possess a high academic English proficiency. However, there are variations in the English proficiency of the instructors. While some possess a high level of expertise, the use of English hinders their ability to engage in a lively classroom interaction. The primary factors that safeguard the quality of instruction are the selection and training of EMI instructors.

N23: “I think the construction of the Nursing EMI teaching crew should be paid sufficient attention to, teachers should be encouraged to upgrade their academic proficiency, go abroad for further study, enhance their teaching ability, and afterwards pass on the knowledge to us.” Therefore, it is imperative that college prioritize the ongoing training of Nursing EMI course instructors.

#### A more international platform provided for students

As a facilitator, the college should provide students with a more international development platform, paying adequate attention to students’ opportunities of educational exchange.

N11: “International exchange activities are important; thus, it would be great if schools provided us more opportunities. College functions as a facilitator and booster, so students are more likely to get a better study platform since we wouldn’t have such access to these opportunities on our own.”

#### Fortifying connection with senior honored students

Students believed that experience-sharing conferences for Nursing EMI students should be held more frequently, and referential experience should be imparted by previous graduates or senior students to the junior EMI students, to help them better adapt to the EMI mode, and simultaneously provide positive feedback to boost their proactivity and self-confidence.

Honored seniors, having gone through similar challenges, can offer empathetic and targeted support that resonates more deeply than institutional guidance. N21 stated: “I think it would be more beneficial to have senior students serve as our guides, as they have also experienced this phase. They are capable of comprehending our challenges and providing assistance in resolving them.”

Beyond adaptation support, juniors also hope to learn from honored seniors’ experiences in long-term goals, to make more informed decisions and avoid common mistakes. N8 articulated this need, noting: “I learned that previous Nursing EMI graduates outperformed their colleagues in overseas study applications, Chinese postgraduate admission tests, and clinical nursing job performance. I’m hoping that senior students may share their knowledge and assist us avoid detours.”

## Discussion

### Nursing EMI improving students’ comprehensive quality but remaining challenging

With the trend of global health and escalating requirements of Nursing undergraduates’ professional English for various settings such as graduation, job application and clinical work, the implementation of Nursing EMI is imperative [26, 27], in spite of the increased academic burden it may bring.

Students in this study generally reported that using English as a second language presented greater challenges in adapting to EMI courses, which is consistent with previous research [28, 29]. Despite investing more time and effort, students often exhibited less satisfactory academic performance compared to their peers studying in their native language, as also reported in other studies [1, 8, 30, 31]. However, a study has reported that students did not encounter substantial language-related difficulties when participating in EMI courses, which may be related to the varying English proficiency thresholds required for admission into different EMI programs [32]. In addition to the challenges caused by language barriers, such as reduced learning efficiency and unremarkable academic performance, students in this study also reported that the heterogeneity of instructional materials was a significant difficulty in the learning process. Similar findings were reported by Anna Williams [33], whose survey of instructors indicated that English instructors often develop their own teaching materials.

While non-native English learners faced various challenges in the learning process of professional courses, students in the EMI program has shown substantial long-term benefits. As students gradually adapted to the demands of EMI, they experienced notable improvements in several areas. These included improved comprehensive ability, greater self-confidence, clearer professional identity and career direction, and enhanced global vision and international competence. Other studies have similarly reported that students enrolled in EMI programs have demonstrated significant gains in multiple competencies [34, 35]. These outcomes reflect not only personal growth but also better preparedness for future academic and professional pursuits.

These findings reveal a dynamic interplay between the challenges and advantages associated with EMI, rather than treating them as isolated or opposing outcomes. The difficulties encountered by ESL students, such as limited comprehension, heightened academic stress, and lower performance, functioned not only as hindrances but also as catalysts for adaptive learning behaviors and cognitive growth. EMI should be understood as a developmental ecology in which challenges and benefits co-evolve.

Admittedly, it is of vital significance to acknowledge alternative explanations. For instance, students enrolled in the EMI program may already possess higher levels of academic motivation or language proficiency, which could predispose them to greater academic gains regardless of the instructional medium. In addition, the observed improvements may also be influenced by the quality of instruction, availability of institutional resources, or the innovative teaching methods (e.g., PBL, flipped classrooms) used in some EMI courses, rather than EMI per se.

### Nursing EMI remaining in the exploratory stage

A standardized pedagogical framework for the Nursing EMI paradigm has not yet been established in China, and several urgent issues remain to be addressed. Students’ suggestions, which have highlighted critical gaps in the current nursing EMI programs, offer targeted and actionable insights for program refinement. Grounded in learners’ firsthand experiences, proposed improvements can be structured across four key domains: Curricular System, Teaching Resources, Faculty Training and Student Support.

In terms of the curricular system, systematically enhancing the quality of Nursing EMI courses requires pedagogical reform centered on student development, learning processes, and educational outcomes. Based on the findings of this study, curriculum optimization should focus on three key areas: clarification of teaching goals, restructuring of course content, and adjustment of assessment strategies. First, clearer articulation of teaching goals is essential to align academic instruction with students’ future career trajectories, whether in domestic clinical settings or international academic environments. Well-defined goals can help guide students’ learning focus and professional identity formation. Second, a more coherent curriculum structure, emphasizing integration of core nursing content and stepwise incorporation of English medical terminology, would improve learners’ academic fluency. Previous research has repeatedly emphasized that language barriers remain a major impediment to EMI effectiveness. For instance, Lin Cen observed in the exploration of a bilingual instructional model in Introduction to Nursing that although students acknowledged the importance of English in professional courses, many lacked the confidence to learn exclusively in English [36], a finding echoed by Vico Chiang et al. [37]. In order to address the problem of students’ backward clinical English level, a university in Hong Kong launched an English support course, which has been considered a great help [38]. Similarly, an additional English training course Academic English was provided by X(referring to a specific University Medical College) to help students with the acquaintance with medical terms and grasp of clinical reasoning. Finally, assessment practices should reflect the distinctive challenges faced by EMI students. Tailored evaluation systems, including formative assessments and adjusted scholarship ranking mechanisms, can ensure fairer academic appraisal.

In addition to curricular reforms, enhancing teaching resources represents another pivotal strategy. Sustainable digital learning platforms should be constructed, through recording bilingual (English/Chinese) instructional videos, and developing banks of clinical scenarios and examination tests. This technological scaffolding helps strengthen students’ ability of analyzing and managing the learning data, facilitating the continuous learning feedback. Furthermore, students in this study emphasized the importance of international exposure. Universities are therefore encouraged to act as facilitators by establishing institutional platforms for exchange and collaboration, enabling learners to expand their global perspective and strengthen their international competitiveness.

Equally important is the investment in faculty training. In this study, students voiced their anticipation that more lecturers with higher English proficiency should be enrolled, which was consistent with Anja Garone et al.’s research [3]. Faculty development and training should be strengthened, through systematic and continuous training in advanced pedagogical approaches and educational technology. At the same time, the team’s expertise in both nursing specialization and English proficiency should be further leveraged, to conduct bilingual analysis and research on core nursing content such as nursing theory and nursing process.

Finally, student support mechanisms must be reinforced, especially through stronger peer networks. Connecting junior EMI students with senior peers and alumni was identified as an effective way to foster adaptation.

### Growth and achievements of nursing EMI program

Under the contemporary circumstances in China, growing numbers of universities in Mainland, China initiate programs using English as an instructional medium, among which the infiltrative and transitional bilingual mode are most common ones [5, 39, 40]. As one of the few universities which provide EMI paradigm in Mainland, China [1, 41], X(referring to a specific University Medical College) has been dedicated to forging a Nursing program conforming to China’s national context as well as possessing the X(referring to a specific University Medical College) hallmark. The EMI teachers received accreditation from a committee composed of English-speaking medical professors and English teachers, and completed an educational reform. Since the establishment in 2013, the Nursing EMI program in X(referring to a specific University Medical College) has trained A number of graduates, with a proportion of 96% engaged in nursing-related work. In total, about 55% further studied for a master’s degree, and 18% went to elite universities such as the University of Washington in the United States, the University of Sydney in Australia. Most of those chose to work were employed in affiliated hospitals of prestigious universities in China such as Zhejiang University and Sun Yat-sen University, with promising career prospects. The Nursing EMI in X(referring to a specific University Medical College) has long adhered to the concept of “international education”, and been actively devoted to developing joint cultivation projects, short-term exchange projects and communication lectures by foreign specialists, as well as hosting or undertaking international seminars, which has provided teachers and students with a superb platform for international academic and teaching communication.

### Nursing EMI program conforming to the internationalization of tertiary education and development trend of global health

China’s Education Modernization 2035 issued by the Central Committee of the Communist Party of China and the State Council [42], as a guideline document for China to establish an educational power in the new era, focuses on the strategic task of “creating a new scheme of education opening up to the outside world” to thoroughly improve the opening-up level of China’s education and international influence. Meanwhile, due to the frequent flow of international population and the increase in the demand for foreign-related medical services, the quality and quantity of international Nursing elites are far from meeting the needs of the new situation [30]. As a hub of Nursing concepts and cultural connections between China and abroad, Nursing EMI program cultivating nursing talents with global vision and international competitiveness, and helping with the gradual integration of domestic and international Nursing, conforming to the developmental trend in the new era, is one of the important ways to actualize the international development of Nursing [43]. How to learn from the international preeminent training system, focus on the quality cultivation of talents, and develop a tertiary Nursing education model as well as system in line with China’s situation remains one of the keys of the conceptual shift and system reform of Nursing education.

## Conclusions

This qualitative study provides preliminary insights into the learning experiences of Chinese undergraduate nursing students enrolled in an EMI program. The findings reveal a threefold structure comprising students’ perceived advantages of EMI, the challenges they encountered during the learning process, and their suggestions for improving EMI implementation.

Several limitations of this study should be acknowledged. First, the study adopted a convenience sampling strategy, and participants were limited to current nursing undergraduates from a single institution, with no involvement of graduates. These sampling characteristics may have introduced self-selection bias. Second, the study focused solely on students’ perspectives, without incorporating insights from faculty members or administrators, which restricts the comprehensiveness of the findings regarding EMI program implementation. Third, data collection relied exclusively on focus group discussions, without triangulation from classroom observations, academic records, or institutional documents, which may limit the credibility of interpretations.

Therefore, the findings should be interpreted as exploratory and context-specific. Future studies are recommended to involve more diverse samples and stakeholders, adopt longitudinal or mixed-method designs, and integrate multiple data sources to validate and extend the current findings. This study serves as an initial step toward understanding the complex dynamics of EMI in nursing education within non-English-speaking contexts.

## Supplementary Information

Supplementary material 1.

## Data Availability

No datasets were generated or analysed during the current study.

## Abbreviations

EMI: English as a Medium of Instruction
FGD: Focus group discussion
HEIs: Higher education institutions
ESL: Second language
GPA: Grade point average
FLM: Flipped learning models
PBL: Problem-based learning

## References

[1] Yang M, O’Sullivan PS, Irby DM, Chen Z, Lin C, Lin C. Challenges and adaptations in implementing an English-medium medical program: a case study in China. BMC Med Educ. 2019;19(1):15. 30626387 10.1186/s12909-018-1452-3 PMC6325837

[2] Pitkajarvi M, Eriksson E, Kekki P. Teachers’ experiences of English-language-taught degree programs within health care sector of Finnish polytechnics. Nurse Educ Today. 2011;31(6):553–7. 21095046 10.1016/j.nedt.2010.10.032

[3] Garone A, Van de Craen P, Struyven K. Multilingual nursing education: nursing students’ and teachers’ interests, perceptions and expectations. Nurse Educ Today. 2020;86:104311. 31841829 10.1016/j.nedt.2019.104311

[4] Kunaviktikul W, Turale S. Internationalizing nursing curricula in a rapidly globalizing world. Nurse Educ Pract. 2020;43:102704. 31991380 10.1016/j.nepr.2020.102704

[5] Liao ML, Yeh CC, Lue JH, Chien CL, Hsu SH, Chang MF. Benefits of a bilingual web-based anatomy atlas for nursing students in learning anatomy. BMC Med Educ. 2022;22(1):341. 35505291 10.1186/s12909-022-03405-8 PMC9064542

[6] Sabbour SM, Dewedar SA, Kandil SK. Language barriers in medical education and attitudes towards Arabization of medicine: student and staff perspectives. East Mediterr Health J. 2012;16(12):1263–71. 24988402 10.26719/2010.16.12.1263

[7] Lahtinen P, Leino-Kilpi H, Salminen L. Nursing education in the European higher education area - variations in implementation. Nurse Educ Today. 2014;34(6):1040–7. 24090615 10.1016/j.nedt.2013.09.011

[8] Macaro E, Curle S, Pun J, An J, Dearden J. A systematic review of English medium instruction in higher education. Lang Teach. 2018;51(1):36–76.

[9] Cots JM. Introduction: English-medium instruction at the University of Lleida, Spain: intervention, beliefs, and practices. In: Doiz A, Lasagabaster D, Sierra JM, editors. English-medium instruction at universities: global challenges. Bristol: Multilingual Matters; 2013. p. 106–28.

[10] Earls CW. Evolving agendas in European English-medium higher education: interculturality, multilingualism and language policy. Basingstoke: Palgrave Macmillan; 2016.

[11] Piller I, Cho J. Neoliberalism as language policy. Lang Soc. 2013;42:23–44.

[12] Nguyen HT, Walkinshaw I, Pham HH. EMI programs in a Vietnamese university: language, pedagogy and policy issues. In: Fenton-Smith B, Humphreys P, Walkinshaw I, editors. English medium instruction in higher education in Asia-Pacific. Dordrecht: Springer; 2017. p. 37–52.

[13] Crawford T, Candlin S. A literature review of the language needs of nursing students who have English as a second/other language and the effectiveness of English language support programmes. Nurse Educ Pract. 2013;13(3):181–5. 23041163 10.1016/j.nepr.2012.09.008

[14] Gupta MM, Deshmukh M, Chari S. Is English language as a medium of instruction a hurdle for first year MBBS teaching learning? Perceptions of students and teachers. Int J Res Med Sci. 2017;5:4195.

[15] Alhamami M, Almelhi A. English or Arabic in healthcare education: perspectives of healthcare alumni, students, and instructors. J Multidiscip Healthc. 2021;14:2537–47. 34552332 10.2147/JMDH.S330579 PMC8450159

[16] Joe YJ, Lee H. Does english-medium instruction benefit students in EFL contexts? A case study of medical students in Korea. Asia Pac Educ Res. 2013;22:201–7.

[17] Alshareef M, Mobaireek O, Mohamud M, Alrajhi Z, Alhamdan A, Hamad B. Decision makers’ perspectives on the language of instruction in medicine in Saudi Arabia: a qualitative study. Health Prof Educ. 2018;4(4):308–16.

[18] Alhamami M. One decade of “English as a medium of instruction” (EMI) in healthcare education. Front Med (Lausanne). 2024;11:1296563. 38487028 10.3389/fmed.2024.1296563 PMC10937345

[19] Park H. English medium instruction and content learning. Engl Lang Linguist. 2007;23(2):257–74.

[20] Mann C, Canny BJ, Reser DH, Rajan R. Poorer verbal working memory for a second language selectively impacts academic achievement in university medical students. PeerJ. 2013;1:e22. 23638357 10.7717/peerj.22 PMC3628612

[21] Horwood C, Mapumulo S, Haskins L, John V, Luthuli S, Tylleskär T, et al. A north-South-South partnership in higher education to develop health research capacity in the Democratic Republic of the Congo: the challenge of finding a common language. Health Res Policy Syst. 2021;19(1):79. 33962628 10.1186/s12961-021-00728-8 PMC8106225

[22] Lucas P, Lenstrup M, Prinz J, Williamson D, Yip H, Tipoe G. Language as a barrier to the acquisition of anatomical knowledge. Med Educ. 1997;31(2):81–6. 9231106 10.1111/j.1365-2923.1997.tb02463.x

[23] Al Zumor AQ. Challenges of using emi in teaching and learning of university scientific disciplines: student voice. Int J Lang Educ. 2019;3:74–90.

[24] Pomat N, Jannok A, Buripakdi A, Wilang JD. Partial EMI nursing program: insights from students and teachers in Thailand. Theory Pract Lang Stud. 2022;12:1386–96.

[25] Johnson JL, Adkins D, Chauvin S. A review of the quality indicators of rigor in qualitative research. Am J Pharm Educ. 2020;84(1):7120. 32292186 10.5688/ajpe7120 PMC7055404

[26] Yang X. The perception of bilingual teaching among nursing students in Western University for nationalities: a qualitative research. J Northwest Univ Natl (Nat Sci Ed). 2019;40(4):88–92.

[27] Gasiorek J, van de Poel K. Language-specific skills in intercultural healthcare communication: comparing perceived preparedness and skills in nurses’ first and second languages. Nurse Educ Today. 2018;61:54–9. 29175688 10.1016/j.nedt.2017.11.008

[28] Starr K. Nursing education challenges: students with English as an additional language. J Nurs Educ. 2009;48(9):478–87. 19645373 10.3928/01484834-20090610-01

[29] Jin J. Understanding silence in problem-based learning: a case study at an English medium university in Asia. Clin Linguist Phon. 2014;28(1–2):72–82. 23895282 10.3109/02699206.2013.813587

[30] Wu M. Focus on the teaching reform of nursing English course and cultivation of international nursing talents. Guide Sci Educ. 2022;2022(17):117–9.

[31] Liu M. Exploration of bilingual teaching mode of foreign related nursing physiology in vocational colleges based on flipped classroom. Theory Pract Innov Entrepren. 2022;5(9):138–40.

[32] Abdeljaoued M. English-medium instruction in Tunisia: perspectives of students. Front Psychol. 2023;14:1112255. 37008855 10.3389/fpsyg.2023.1112255 PMC10063842

[33] Williams A, Stevens JR, Anderson R, Bogren M. Challenges and opportunities of English as the medium of instruction in diploma midwifery programs in Bangladesh: a mixed-methods study. BMC Med Educ. 2024;24(1):523. 38730449 10.1186/s12909-024-05499-8 PMC11088132

[34] Caputi L, Engelmann L, Stasinopoulos J. An interdisciplinary approach to the needs of non-native-speaking nursing students: conversation circles. Nurse Educ. 2006;31(3):107–11. 16708033 10.1097/00006223-200605000-00006

[35] Amaro DJ, Abriam-Yago K, Yoder M. Perceived barriers for ethnically diverse students in nursing programs. J Nurs Educ. 2006;45(7):247–54. 16863104 10.3928/01484834-20060701-03

[36] Lin C, Lai X, Qian X. Practice and evaluation of progressive bilingual teaching in the introduction to nursing curriculum. Health Vocat Educ. 2023;41(8):46–9.

[37] Chiang V, Crickmore BL. Improving English proficiency of post-graduate international nursing students seeking further qualifications and continuing education in foreign countries. J Contin Educ Nurs. 2009;40(7):329–36. 19639855 10.3928/00220124-20090623-03

[38] Gardner J. Barriers influencing the success of racial and ethnic minority students in nursing programs. J Transcult Nurs. 2005;16(2):155–62. 15764639 10.1177/1043659604273546

[39] Cai C, Zhang C, Wang Y, Xiong L, Jin Y, Jin C. Nursing students’ satisfaction with bilingual teaching in nursing courses in China: a meta-analysis. Nurse Educ Today. 2016;44:51–8. 27429329 10.1016/j.nedt.2016.05.014

[40] He W, Xu Y, Zhu J. Bilingual teaching in nursing education in China: evolution, status, and future directions. Nurs Health Sci. 2011;13(3):371–7. 21749591 10.1111/j.1442-2018.2011.00623.x

[41] Huang S, Zhuang W, Huang H, et al. Comparing domestic and overseas all-English teaching experience and innovating medical education mode: taking Shantou University Medical College as an example. Chin Med Educ Technol. 2020;34(3):388–91.

[42] Zhang M, Liu B. The policy trends of creating a new pattern of opening-up of education in the new era: based on the interpretation of “China’s education modernization 2035.” China Educ Technol. 2020;2020(1):25–32.

[43] Garone A, Van de Craen P. The role of language skills and internationalization in nursing degree programmes: a literature review. Nurse Educ Today. 2017;49:140–4. 27940365 10.1016/j.nedt.2016.11.012

