# Supplementary material for: Understanding the learning experience of Chinese nursing students in an English-medium instructional program: growth and challenges
Source: BMC Med Educ. 2025 Oct 9;25:1392. doi: 10.1186/s12909-025-07966-2 (PMC12512413; doi:10.1186/s12909-025-07966-2)
Supplement: Supplementary file 1 — Supplementary material 1. [file 12909_2025_7966_MOESM1_ESM.docx]

**Interview Guide**

**Background Information**

The aim of this interview is to understand your learning experiences and viewpoints regarding the Nursing EMI (English as a Medium of Instruction) program. Your feedback will contribute to the improving teaching approaches and enhancing student learning experiences. All information will be kept confidential, and your personal privacy will be fully protected.

**Opening Questions**

**Section 1: Learning Experience** (What kind of experience do you think it is to learn in the Nursing EMI program?)

1. Why did you join the Nursing EMI program in the first place?

2. Do you feel worried or motivated when teachers utilize English as a teaching medium? In what period did you adapt to studying nursing in English? What helped you gradually adapt to this learning model?

3. What are your learning strategies for Nursing EMI program?

4. Does the Nursing EMI program meet your expectations? What kind of Nursing EMI program is originally expected? Which part of the program does not meet your expectation?

5. What do you think of Nursing EMI Program lecturers? What role do you think foreign lecturers have been performing in Nursing EMI program?

**Section 2: Competency Development** (Through Nursing EMI learning, in what aspects you got improved?)

10.In your mind, what have you gained from Nursing EMI program? what aspects of abilities were improved?

12. In your mind, what courses or modules in the Nursing EMI program can best cultivate students’ abilities?

15. From freshman to junior, what changes happened in your professional identity?

**Section 3: Learning Challenges** (In your mind, what are the most difficult parts of Nursing EMI learning?)

17. Does studying nursing in English have any effect on your psychological stress levels? How do you relieve the stress?

19. What are your learning weaknesses as compared to non-EMI nursing students?

**Section 4: Suggestions for Improvement** (What aspects of the Nursing EMI program do you think can be improved?)

20. In your mind, what are the challenges in carrying out Nursing EMI program in our school? what is the significance of Nursing EMI program in Mainland China?

23. Which aspects should we be emphasizing to advance the Nursing EMI program?

**Closing Questions**

24. What more Nursing EMI learning experiences would you like to discuss?

25. Thank you very much for your time and valuable insights.

**Ethical and Consent Statement**

Participation in this interview is entirely voluntary. You can withdraw at any time or choose not to answer any questions you do not wish to answer. Your responses will be anonymized and used solely for academic research purposes.
